# Other considerations than: how much will universal health coverage cost?

**DOI:** 10.2471/BLT.19.238915

**Published:** 2019-11-22

**Authors:** Sarah L Barber, Sheila O'Dougherty, Lluis Vinyals Torres, Tsolmongerel Tsilaajav, Paul Ong

**Affiliations:** a World Health Organization Centre for Health Development, IHD Center Building 9F, 1-5-1 Wakinohama-Kaigandori, Chuo-Ku, Kobe, 651-0073, Japan.; b Abt Associates, Washington DC, United States of America.; c World Health Organization South-East Asia Regional Office, New Delhi, India.

## Abstract

Globally, countries have agreed to pursue the progressive realization of universal health coverage (UHC) and there is now a high level of political commitment to providing universal coverage of essential health services while ensuring that individuals are financially protected against high health spending. The aim of this paper is to help policy-makers think through the progressive realization of UHC. First, the pitfalls of applying global normative expenditure targets in estimating the national revenue required for UHC are discussed. Then, several recommendations on estimating national revenue are made by moving beyond the question of how much UHC will cost and focusing instead on the national health-care reforms and policy choices needed to progress towards UHC. In particular, costing exercises are recommended as a tool for comparing different service delivery options and investment in data infrastructure is recommended for improving the information needed to identify the best policies. These recommendations are intended to assist health policy-makers and international and national agencies who are developing country plans for the progressive realization of UHC.

## Résumé

À l'échelle mondiale, les pays sont convenus de poursuivre la réalisation progressive de la couverture sanitaire universelle, et l'on observe désormais un fort niveau d'engagement politique en faveur de la couverture universelle des services de santé essentiels en veillant à ce que les individus soient financièrement à l'abri de toute dépense de santé élevée. L'objectif de cet article est d'aider les responsables politiques à effectuer un examen minutieux en vue de la réalisation progressive de la couverture sanitaire universelle. Pour commencer, nous examinons les écueils liés à l'application d'objectifs de dépenses normatifs mondiaux au moment d'estimer le revenu national requis pour la couverture sanitaire universelle. Nous formulons ensuite plusieurs recommandations concernant l'estimation du revenu national, en dépassant la question du coût de la couverture sanitaire universelle pour nous concentrer sur les réformes nationales en matière de soins de santé et sur les choix politiques nécessaires pour faire progresser la couverture sanitaire universelle. Nous recommandons notamment de procéder à des exercices d'établissement des coûts pour comparer différentes options de prestation de services et d'investir dans des infrastructures de données pour améliorer les informations nécessaires à l'identification des meilleures politiques. Ces recommandations visent à aider les responsables des politiques de santé et les organismes internationaux et nationaux qui élaborent des plans nationaux pour la réalisation progressive de la couverture sanitaire universelle.

## Resumen

A nivel mundial, los países han acordado procurar la realización progresiva de la cobertura sanitaria universal (universal health coverage, UHC) y ahora existe un alto nivel de compromiso político para proporcionar una cobertura universal de los servicios sanitarios esenciales, al tiempo que se garantiza la protección financiera de las personas frente a los elevados gastos sanitarios. El objetivo de este documento es ayudar a los responsables de formular políticas a pensar en la realización progresiva de la UHC. Primero, se discuten las trampas en la aplicación de las metas globales de gastos normativos al estimar los ingresos nacionales requeridos para la UHC. Luego, se hacen varias recomendaciones sobre la estimación de los ingresos nacionales al ir más allá de la cuestión de cuánto costará la UHC y enfocarse en cambio en las reformas nacionales de salud y en las opciones de políticas necesarias para progresar hacia la UHC. En particular, se recomiendan ejercicios de cálculo de costos como herramienta para comparar diferentes opciones de prestación de servicios y se recomienda invertir en infraestructura de datos para mejorar la información necesaria con el fin de identificar las mejores políticas. Estas recomendaciones tienen por objeto ayudar a los responsables de formular políticas de salud y a los organismos internacionales y nacionales que están elaborando planes nacionales para la realización progresiva de la UHC.

## ملخص

وافقت البلدان على مستوى العالم على السعي نحو التنفيذ التدريجي للتغطية الصحية الشاملة (UHC)، وهناك الآن مستوى عالٍ من الالتزام السياسي نحو توفير تغطية شاملة للخدمات الصحية الأساسية، مع ضمان الحماية المالية للأفراد ضد ارتفاع الإنفاق على الصحة. الهدف من هذه الورقة هو مساعدة صناع السياسة على التفكير من خلال التنفيذ التدريجي للتغطية الصحية الشاملة. أولاً، تمت مناقشة مخاطر تطبيق أهداف الإنفاق المعياري العالمي في تقدير الدخل الوطني المطلوب للحصول على التغطية الصحية الشاملة. بعد ذلك، يتم تقديم العديد من التوصيات الخاصة بتقدير الدخل الوطني، وذلك عن طريق التحرك لما بعد موضوع تكلفة الرعاية الصحية الشاملة، والتركيز بدلاً من ذلك على إصلاحات الرعاية الصحية الوطنية وخيارات السياسة المطلوبة للتقدم نحو التغطية الصحية الشاملة. ويوُصى على وجه الخصوص بتدريبات التكاليف كأداة لمقارنة الخيارات المختلفة لتقديم الخدمات، كما يوصى بالاستثمار في البيانات بهدف تحسين المعلومات المطلوبة لتحديد أفضل السياسات. الهدف من هذه التوصيات هو مساعدة واضعي السياسات الصحية، والهيئات الدولية والوطنية، الذين يقومون بوضع خطط الدولة للتنفيذ التدريجي للتغطية الصحية الشاملة.

## 摘要

在全球范围内，各国已同意逐步实现全民健康覆盖 (UHC)，目前，各国在政治上高度致力于提供基本健康服务的全民覆盖范围，同时确保个人免受高额医疗支出的经济负担。本文旨在帮助政策制定者思考如何逐步实现全民健康覆盖 (UHC)。首先，讨论采用全球规范性支出目标来估算实现全民健康覆盖 (UHC) 所需的国家税收的困难。然后，对估算国家税收提出若干建议，这些建议不仅围绕全民健康覆盖 (UHC) 的成本问题，而且把重点放在推进全民健康覆盖 (UHC) 所需的国家医疗改革和政策选择上。特别是，建议将成本计算工作作为比较不同服务交付选择的工具，并建议对数据基础架构进行投资，以改进确定最佳政策所需的信息。这些建议旨在协助健康政策制定者以及正在制定逐步实现全民健康覆盖计划的国内外机构。

## Резюме

Страны мира пришли к соглашению о продолжении последовательной реализации программы всеобщего охвата услугами здравоохранения (ВОУЗ). В настоящее время обеспечение всеобщего охвата основными услугами здравоохранения с одновременным созданием финансовой защиты отдельных категорий лиц от высоких расходов на медицинское обслуживание получает значительную политическую поддержку. Цель данного документа — помочь лицам, формирующим политику, продумать процесс последовательной реализации программы ВОУЗ. В нем обсуждаются возможные скрытые проблемы внедрения глобальных нормативов целевых расходов при оценке национального дохода, необходимого для обеспечения всеобщего охвата услугами здравоохранения. Документ также содержит несколько рекомендаций по оценке национального дохода посредством не только рассмотрения вопроса о том, сколько будет стоить обеспечение ВОУЗ, но и заострения внимания на национальных реформах в сфере здравоохранения и изменениях в политике, необходимых для достижения прогресса в обеспечении всеобщего охвата услугами здравоохранения. В частности, рекомендуется использовать расчет стоимости в качестве инструмента сравнения различных вариантов предоставления услуг, а также инвестиции в инфраструктуру анализа данных для повышения качества информации, необходимой для разработки наиболее эффективных политик. Такие рекомендации предназначены для оказания содействия лицам, формирующим политику в сфере здравоохранения, а также международным и национальным ведомствам, разрабатывающим планы последовательной реализации программы ВОУЗ на уровне стран.

## Introduction

In 2015, United Nations’ Member States agreed to pursue the progressive realization of universal health coverage (UHC) as part of their commitment to the sustainable development goals. The World Health Organization (WHO) defines UHC as providing all people and communities with the promotive, preventive, curative, rehabilitative and palliative health services they need, of a sufficient quality, while also ensuring that use of these services does not result in financial hardship.^1^ Having made the political commitment, policy-makers in national ministries of health and finance are now asking the reasonable question of how much UHC will cost. To help policy-makers think through this question for their own countries, we first discuss the importance of reframing the question such that the focus is less on global normative expenditure targets and more on the national policy choices and reforms needed to expand coverage and provide financial protection to all. Then we make recommendations that may be useful for estimating the national revenue required to move progressively towards UHC in individual countries. These recommendations are intended for health policy-makers and international and national agencies who are developing country plans for the progressive realization of UHC.

## The pitfalls of global targets

In answering the question, “How much will UHC cost?,” national policy-makers frequently start with a straightforward comparison between current per-capita funding for health in their own country and global targets, which range from 54 to 86 United States dollars (US$) per person annually.^2^ This approach is problematic for several reasons.

First, one needs to recall that global normative expenditure targets were developed primarily for advocacy purposes. They serve to highlight the importance of health as a contributor to national development and to generate political commitment. Although global targets may be useful for mobilizing donor funds and for identifying countries that need financial assistance, they were not intended to be used for developing national revenue estimates or for national planning. In situations where health budgets are limited, comparing current spending with global targets can lead to unrealistic estimates. For example, low-income countries, such as Bangladesh, which currently spends less than US$ 30 per person annually on health,^3^ would conclude they need to double or triple spending. This conclusion is an unrealistic starting point for discussions between the health ministry and the finance ministry.

Second, focusing on global normative targets can lead to the erroneous assumption that UHC is a target to achieve, a threshold or a single fixed outcome that does not change over time. Instead, UHC is an objective that must be pursued continuously through reform of, and investment in, the health system.^4^ Although targets have been established to monitor service coverage and financial protection, these targets should not be confused with the progressive realization of UHC. Mongolia, for example, has for 15 years implemented reforms that aim to reduce high out-of-pocket expenditure on health.^5^ Similarly, all countries can strive to implement reforms that promote universal access, higher quality and financial protection, regardless of the resources they dedicate to health.^6^

Third, the concept of a global normative target suggests that all countries need to spend a defined amount on health to achieve the same outcomes. There is evidence that high public health spending can result in better service coverage and financial protection.^4^ However, the production of health is influenced by important factors that are specific to individual countries, such as the labour cost of health-care workers, the capital cost of buildings, the price of medical products and health services, and insurance arrangements.^7^ How national resources are managed also matters. In 2016, WHO found that the performance of different countries in improving coverage and financial protection varied widely, regardless of whether their health budget was low or high (i.e. over US$ 520 per person annually).^4^ For any given level of health spending, countries vary in health performance and achievements. Therefore, all countries have room for progress towards UHC. The question is less about achieving a spending target and more about what can be done with the resources available. For example, in 2017 the United States of America spent 18% of its gross domestic product on health, but millions of its citizens were without access to essential health services or financial protection.^8^

Fourth, global normative targets tend to focus attention on the funding gap alone. This focus has led some policy-makers and donors to suggest that private financing could fill the gap left by the limited fiscal capacity of a country’s government.^9^ Using private financing to fill funding gaps is problematic if there is no overall vision of how private funding fits with the broader goals for UHC and financial protection. One should note that no country has attained full coverage or financial protection for their inhabitants by relying mainly on private financing or voluntary insurance. Rather countries have made progress through a mix of funding sources, including general government revenue and mandatory insurance.^10^ Private financing sources can indeed generate substantial funding but, paradoxically, a high level of spending alone might not help realize UHC; it could even increase inequities.^11^ In some cases, a high level of private spending on health can divert scarce human resources to the privately insured at the expense of the rest of the population. In South Africa, for example, health expenditure from private voluntary insurance was equivalent to 4% of the country’s gross domestic product in 2016.^3^ However, as the privately insured accounted for only 16% of the population, this high level of expenditure did not benefit everyone equally.^12^ In fact, private spending moved South Africa further from UHC by increasing resource inequities between the public and private sectors.

## Recommendations

Moving beyond the question of how much UHC will cost enables national policy-makers and health ministries to focus on the policy choices needed to accelerate progress towards UHC. Here, we make several recommendations that may be useful for estimating the national revenue required to move progressively towards UHC in different settings. These recommendations focus on the information needed to engage constructively with national health and finance authorities on the reforms essential for making progress towards UHC.

### Cost accounting exercises

In striving to accelerate progress towards UHC, tough policy choices need to be made on how services are provided, which levels of the health-care system should be involved in providing those services, the cost of the services and the prices that need to be paid for them. Here, the relevant question is, “How much progress can we make at different levels of funding?” We must determine what it will take to cover the entire population with a specified package of benefits under different funding and service delivery scenarios. Consequently, we must undergo a shift in thinking, to see UHC as an operational rather than a political construct. 

Cost accounting exercises have limitations because cost, like UHC, is not a fixed point but a function. Current health expenditure reflects what has been achieved with the existing health system capacity and level of utilization at a single point in time. Such expenditure embodies inefficiencies within the health system, such as low productivity, excess capacity and an inappropriate mix of inputs into the system. Given that inefficiency is common, adding more resources alone would not be expected to result in a linear improvement in performance. Indeed, relying on historical service unit costs, which are based on existing service delivery models and utilization patterns, can be misleading and can even hamper progress. Moreover, one core objective of UHC is to address underlying inefficiencies and, thereby, accelerate progress.

Nevertheless, cost accounting exercises can be very useful if they provide information about the underlying cost structure of service delivery and illustrate the impact of decisions about how health services can be delivered.^13^ Cost studies can model a range of scenarios that make different assumptions about prices or the impact of incentives or that consider various service delivery configurations and levels of service use.

Recent health-care reforms in China, for example, aimed to strengthen health-care provision at county, township and village levels by encouraging patients to seek essential health services at the primary care level rather than from specialists in referral hospitals.^14^ In this instance, a costing exercise could demonstrate that the cost of hospital-based service delivery with fee-for-service payments would be higher than the cost of primary care service delivery with a payment mechanism that involved a budget cap. Costing different scenarios can identify the policy choices that must be made to attain the broader health systems goals of high coverage, efficiency and good quality. In this way, costing exercises can aid decision-making about policy options. Moreover, costing exercises can help identify the investments in infrastructure, resources and payment mechanisms needed to change the service delivery model such that care shifts from hospitals to primary care facilities. This approach may be particularly useful in evaluating reforms of the health-care workforce, which is a key constraint in many settings. Recruiting and deploying the health-care workforce for the progressive realization of UHC involves long-term investment and a multisectoral strategy that will take many years to implement.^15^

#### Using costing exercises

Many countries have developed health sector plans with detailed benefits packages and have estimated the resources required. In some cases, cost estimates have been unrealistic and the projected gaps in funding could not feasibly be closed over the short or medium term.^16^ In Ghana, for example, a costing exercise on the country’s Medium-Term Development Plan for 2010 to 2013 identified a funding gap that required a 113% increase in the government’s health budget.^16^ Politically, such findings can undermine efforts to accelerate progress towards UHC as they imply that large increases in general revenue are needed immediately.

Frequently, costing exercises for health programmes have used a bottom-up approach that has important methodological limitations. For example, aggregating cost estimates for individual services typically leads to a highly inflated estimate of total cost that almost always exceeds the upper bound of the resources available. Moreover, complex modelling that uses weak or inaccurate data can give policy-makers a false impression that the outcomes are more robust than justified.^13^

What to include in a costing exercise and for what purpose need to be well defined. Interpreting the costs of specific health services (e.g. for malaria, maternal health, family planning or human immunodeficiency virus infection) can be difficult, especially at the primary care level, because of the complexity of separating the costs of labour and supplies in facilities where a few health workers care for all patients.^13^ In addition, costing exercises are also affected by inefficient service delivery structures and unpredictable input prices. Then, there are other complicating factors unrelated to accounting, such as, not knowing where patients will access services, the difficulty of separating costs included or excluded from benefits packages, and variations in treatment between practitioners. Consequently, efforts to cost an entire benefits package can be long, confusing and inaccurate.

In contrast, costing a specific step in, or element of, a broader reform process can be valuable, particularly when directly tied to the sequencing of reforms. For example, cost accounting can be useful for setting provider payment rates and for evaluating investments in service delivery improvements. In determining provider payment rates, cost accounting focuses on the relative cost of different types of service output (e.g. simple malaria treatment versus complex cardiovascular inpatient care) and can produce an average cost per service across a group of facilities by apportioning administrative and ancillary costs to the final service output. These relative costs are generally stable for 3 to 5 years and are not influenced by the total budget available, which is based on an annual political decision and results in a base rate or average payment per service.

In Kyrgyzstan, the approach of costing individual elements of health-care reform contributed to the development of a case-based hospital payment system that drove restructuring and efficiency gains.^17^ The approach resulted in policies that allowed facilities to retain savings, matched payments to the services provided and enabled better data collection for the Kyrgyz single-payer system. In this way, cost accounting was used to evaluate specific policy options on the sequencing of reforms. In addition, by improving cost data, this approach also contributed to the development of a benefits package related to the level of service provided by primary care and referral facilities (rather than to a long list of approved services and procedures). Over time and by using ever-improving cost data, the benefits package was adjusted to achieve minimum standards, this improved equity of access by providing comparable funding in rural and urban areas.

### Investing in data infrastructure

Investing in data infrastructure and increasing the availability of good-quality data are critical parts of the health-care reforms needed to move progressively towards UHC. Data can be used to monitor progress in implementing reforms and the availability of accurate data is important for calculating costs and prices. Many countries that have undertaken reforms have also invested in data collection systems to estimate input costs, output volumes and outcomes. In practice, countries can prioritize items that involve large expenditures and data that are feasible to collect; detailed information that is difficult to collect and does not improve the quality of the results can be omitted. Focusing on only essential data can avoid spending time collecting extra information that does not inform a costing analysis.^13^

On the other hand, a lack of data has not prevented countries from implementing reforms in financing. Skeletal data sets can be generated from information that is already available and overall expenditure can be capped through strategic purchasing arrangements. Specifying minimum data sets and putting processes in place will continually improve the information available for decision-making.^13^ One example is the National Health Insurance Scheme in India that was designed for 500 million of the country’s poorest people. As the scheme was established in a very short time, the government set reimbursement rates using available information while also putting into place data collection systems with a review mechanism that enabled these systems to be modified and improved over time.^18^

Careful sequencing of health-care reform implementation can create a dynamic in which the first step makes the second inevitable. For example, estimating the cost of a benefits package does not directly lead to more efficient purchasing of health services or to overcoming the barriers presented by public finance management. However, shifting to an output-based payment system tends to strengthen and harmonize both information and operating systems, thereby improving data and the accuracy of costing exercises. Generally, data that are directly linked to a payment system (and therefore to financial management and audit mechanisms) are more reliable.^19^ In addition, a comparison with expenditure levels and reforms in other countries with similar income levels or in the same region may be helpful in discussions with policy-makers.

## Conclusions

All countries face the ongoing challenge of ensuring that their whole population has access to essential, good-quality health care and is protected against high out-of-pocket spending on health. This paper has highlighted the pitfalls of using global normative targets to produce national revenue estimates. We urge national policy-makers to focus their efforts on the health-care reforms needed rather than on single cost estimates. In practice, the realization of UHC tends to be incremental: service coverage is extended through gradual increases in revenue, health delivery systems are strengthened and efficiency improves. Ultimately, how much UHC will cost depends on the way it is designed and implemented. In reframing the question from “How much?” to “How?,” countries can focus on the health-care reforms, service delivery models and investment needed to provide the foundations for better health among the whole population.
